# Hybrid Assistive Limb Treatment for the Shoulder and Elbow Joints Enabled Recovery from Chronic-Phase Severe C5 Palsy Following Cervical Spine Surgery

**DOI:** 10.3390/jcm14217520

**Published:** 2025-10-23

**Authors:** Yuichiro Soma, Shigeki Kubota, Hideki Kadone, Yukiyo Shimizu, Seioh Ezaki, Yasushi Hada, Yoshiyuki Sankai, Masashi Yamazaki

**Affiliations:** 1 Department of Orthopaedic Surgery, Institute of Medicine, University of Tsukuba, Tsukuba 305-8575, Japan; 2 Division of Regenerative Medicine for Musculoskeletal System, Institute of Medicine, University of Tsukuba, Tsukuba 305-8575, Japan; 3 Ibaraki Prefectural University of Health Sciences, Ibaraki 300-0394, Japan; 4 Center for Innovating Medicine and Engineering (CIME), University of Tsukuba, Tsukuba 305-8575, Japan; 5 Department of Rehabilitation Medicine, Institute of Medicine, University of Tsukuba, Tsukuba 305-8575, Japan; 6 Department of Orthopaedic Surgery, Ichihara Hospital, Tsukuba 300-3253, Japan; 7 Faculty of Systems and Information Engineering, University of Tsukuba, Tsukuba 305-8575, Japan

**Keywords:** exoskeleton device, C5 palsy, cervical spine surgery, shoulder joint, elbow joint

## Abstract

Postoperative C5 palsy is a common complication of cervical spine surgery. Inadequate recovery from C5 palsy can result in significant impairment of activities of daily living. However, no effective treatment has been established for persistent cases. In the present report, we describe a novel therapeutic approach using the Hybrid Assistive Limb (HAL) in a patient with severe, prolonged postoperative C5 palsy. The patient was a 46-year-old man who developed severe right C5 palsy following cervical spine surgery performed 41 months earlier. Despite undergoing conventional rehabilitation, no improvement was observed, and the muscle strength of the right deltoid and biceps remained at manual muscle testing (MMT) grade 2. HAL training, using both shoulder and elbow devices, was initiated at our institution. Training was conducted once weekly for a total of 106 sessions over 21 months. At baseline, the right shoulder range of motion was limited to 50° in flexion and 35° in abduction. With HAL-assisted training, flexion improved to 150° and abduction improved to 95° by the final (106th) session and further increased to 165° and 170°, respectively, at long-term follow-up. Deltoid strength, assessed using handheld dynamometry, increased from 3.5 Nm/kg at baseline to 28.5 Nm/kg after training. In this case, a long-term therapeutic program incorporating shoulder and elbow HAL training successfully improved severe and prolonged postoperative C5 palsy to a functionally useful level. This case highlights the potential effectiveness of HAL therapy for treatment-resistant postoperative C5 palsy.

## 1. Introduction

Postoperative C5 palsy is a frequent complication of cervical spine surgery, manifesting in de novo or exacerbated muscle weakness primarily at the C5 region [1]. Sudden occurrence of motor loss in the deltoid and biceps muscles, corresponding to the C5 myotome, results in difficulties in the elevation of the arm and the flexion of the elbow joint. Previous studies have shown that the occurrence of C5 palsy after cervical spine surgery is 5–14%. As for its prognosis, more than 60% of post-operative C5 palsy patients achieve complete recovery from motor loss [1,2,3,4]. In some patients, however, recovery from C5 palsy is incomplete, and dysfunction of the upper limb persists. C5 palsy causes a significant impediment to daily life, associated with increased healthcare costs and diminished quality of life [5,6]. Despite its impact, there are currently no established guidelines for postoperative rehabilitation in cases of C5 palsy.

Robotic-assisted rehabilitation devices, including exoskeletons and end-effector systems, are increasingly being developed using user-centered approaches to maximize safety, comfort, and therapeutic effectiveness. A recent study on an experimental mechatronic system for gait rehabilitation emphasized the importance of incorporating feedback from potential users during the development phase, demonstrating that usability assessments—including interface design, comfort, and user satisfaction—can help bridge the gap between developer expectations and user needs [7]. These considerations are equally relevant for upper-limb assistive devices, where patient engagement and active participation are critical for effective motor recovery. The Hybrid Assistive Limb (HAL) is a wearable extraskeletal robot developed at our institute [8]. The HAL possesses a specific bioelectrical signal sensor that enables it to support voluntary exercise. Previously, we applied a single-joint HAL for the elbow flexion and extension exercise in a patient with post-operative C5 palsy, and confirmed its safety and feasibility [9]. Subsequently, we developed a shoulder rehabilitation system with the single-joint HAL [10]. The system allows the HAL to detect muscle action potential from the deltoid and assists voluntary shoulder abduction exercises.

In our previous studies, we applied the shoulder HAL for the treatment of patients with severe C5 palsy during the acute post-operative phase [10]. Shoulder abduction training with HAL demonstrated the improvement in their shoulder joint function. Subsequently, we used shoulder HAL training in a patient with delayed recovery from post-operative C5 palsy, in whom severe C5 palsy persisted for 7 months post-operatively, and achieved complete recovery of his shoulder function [11].

In this study, we applied the HAL treatment for the shoulder and elbow joints in a patient with C5 palsy, in whom severe motor loss persisted for 41 months following cervical spine surgery. Conventional rehabilitation offered no realistic prospect of improvement in this case. However, after a total of 106 sessions of HAL treatment, the patient ultimately regained upper limb elevation and elbow flexion. In this paper, we present the detailed treatment course of HAL therapy administered in this case and discuss whether HAL therapy is effective for severe and prolonged post-operative C5 palsy, which is often difficult to treat.

## 2. Case Presentation

### 2.1. Medical History

**Figure 1:**
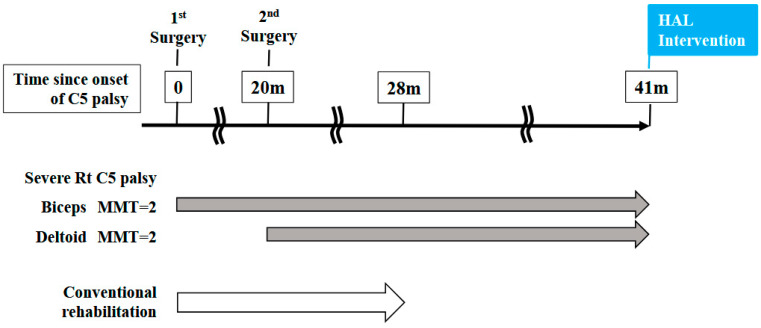
Time course from the onset of C5 palsy to the initiation of HAL treatment in this case.

**Figure 2:**
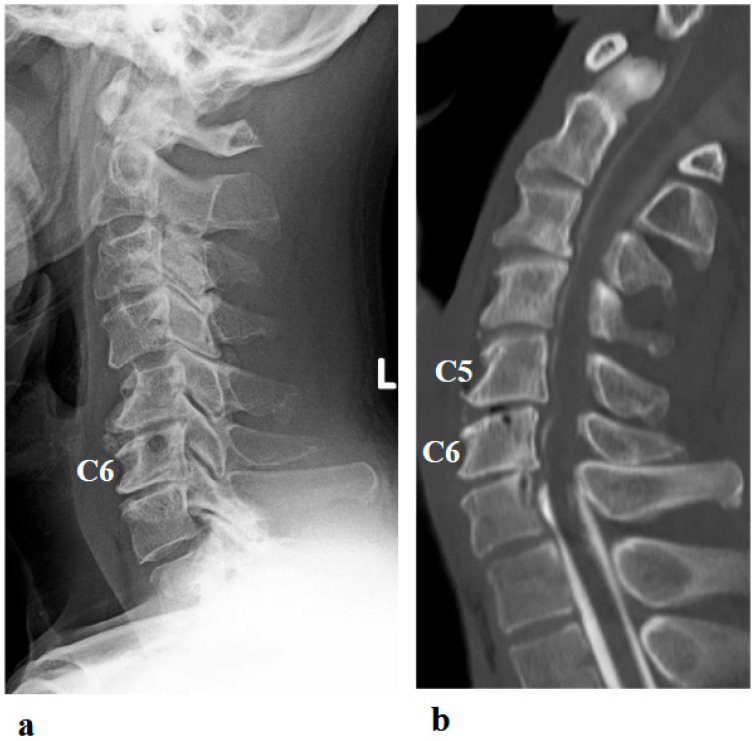

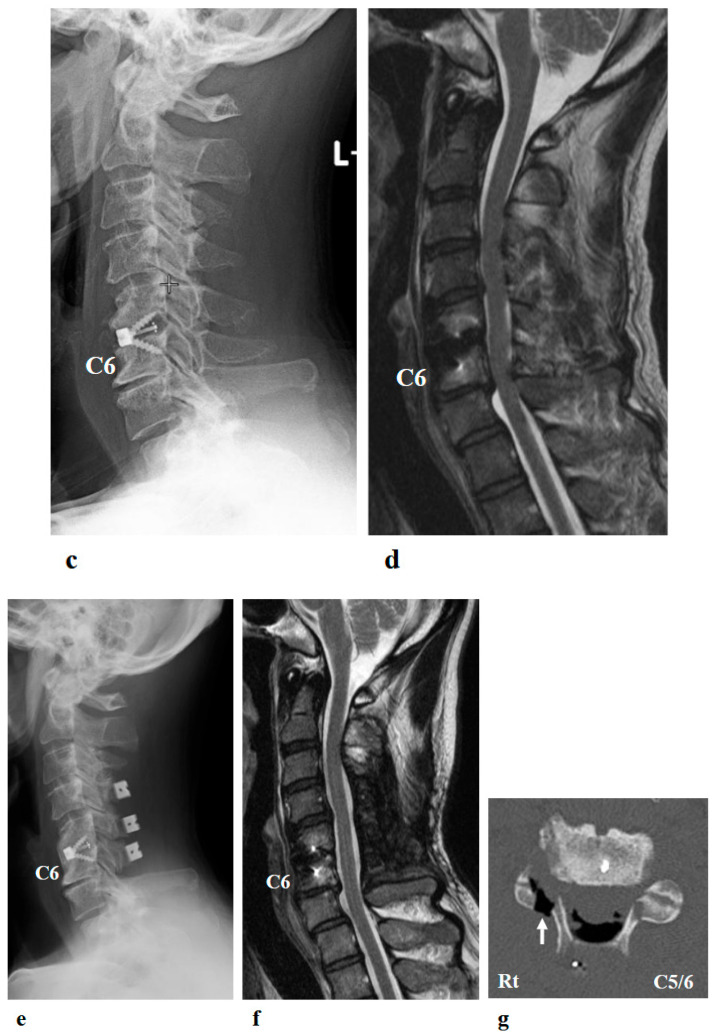
Preoperative (**a**,**b**) and postoperative (**c**,**d**) images following the first surgery, as well as postoperative images from the second surgery (**e**–**g**). (**a**) Lateral cervical spine X-ray showing narrowing of the intervertebral disc space and osteophyte formation at C5–C6 and C6–C7. (**b**) Reconstructed mid-sagittal CT image after myelography revealing spinal cord compression at C5–C6 and C6–C7, and posterior displacement of the C5 vertebral body. (**c**) Postoperative lateral cervical spine X-ray after anterior decompression and fusion at C5–C6. (**d**) Mid-sagittal cervical MRI demonstrating persistent spinal cord compression from C3–C4 to C6–C7. (**e**) Postoperative lateral cervical spine X-ray after C4–C6 laminoplasty and partial laminectomy at the caudal aspect of C3 and the cranial aspect of C7. (**f**) Mid-sagittal cervical MRI showing adequate spinal cord decompression from C3–C4 to C6–C7. (**g**) Axial CT image at the C5–C6 level showing adequate right C5–C6 foraminotomy (arrow).

A 46-year-old man presented to the outpatient clinic of our hospital with a chief complaint of right-sided C5 palsy that had persisted for 41 months following cervical spine surgery (Figure 1). At the age of 43, he noticed a decrease in grip strength in his right hand and numbness in his right upper limb, and visited another hospital. Examinations at that hospital revealed that his initial symptoms were caused by cervical spondylotic myelopathy due to spinal cord compression from degenerative cervical vertebrae (Figure 2a,b). Based on the diagnosis, at that hospital, he underwent anterior decompression and fusion surgery at the C5–C6 level (Figure 2c). Prior to initiation of HAL therapy at our hospital, his neurological assessment was as follows: Numerical Rating Scale for pain = 4, American Spinal Injury Association (ASIA) motor score = 97, and ASIA sensory score = 108.

Postoperatively, although grip strength in his right hand slightly improved, he became aware of weakness in his right elbow flexion. He was unable to flex his elbow independently in both standing and sitting positions, and manual muscle testing (MMT) of his right biceps was grade 2. Although he felt weakness in his deltoid muscle, he was able to elevate his right upper limb (MMT = 3). He was diagnosed with post-operative C5 palsy. Although he received standard rehabilitation at the hospital for C5 palsy, no improvement was observed in the strength of the right biceps and deltoid muscles.

Postoperative imaging revealed spinal cord compression extending from C3–C4 to C6–C7 (Figure 2d). Based on the imaging findings, inadequate spinal cord decompression from the initial surgery was diagnosed, and he underwent an additional cervical spine surgery at the same hospital 20 months later. The second surgery consisted of C4–C6 laminoplasty and partial laminectomy at the caudal aspect of C3 and the cranial aspect of C7 (Figure 2e). During the procedure, surgical intervention for post-operative C5 palsy was concurrently undertaken, specifically a right C5–C6 foraminotomy.

Two days after the second surgery, he experienced pain extending from his neck to his right shoulder, followed by difficulty in elevating his right upper limb. Postoperative MRI demonstrated adequate spinal cord decompression with no obvious abnormal findings (Figure 2f). Post-operative CT confirmed the right C5–C6 foraminotomy was appropriately performed (Figure 2g, arrow).

He was diagnosed with worsening of postoperative right-sided C5 palsy and underwent conventional rehabilitation for the impairment of his right upper limb for eight months after the second surgery at the same hospital. However, despite this rehabilitation, his difficulty in elevation of his right arm and flexion of his right elbow persisted. Forty-one months after the onset of C5 palsy, he presented to our hospital for HAL treatment of the shoulder and elbow joints.

Surgical reinnervation procedures, such as nerve transfer, were considered by the orthopedic surgeons and discussed with the patient. However, given the chronicity of the patient’s C5 palsy and the uncertain potential for recovery, he declined surgical intervention and instead elected to undergo HAL-based rehabilitation.

### 2.2. Physical Findings at Initial Presentation

**Figure 3:**
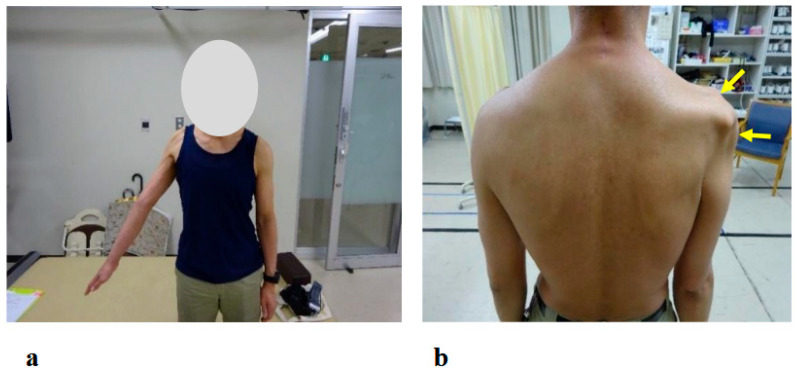
Photographs showing motor paralysis and muscle atrophy of the right shoulder at the initial visit. (**a**) Active right shoulder abduction was limited to 35 degrees. (**b**) Marked muscle atrophy around the right shoulder was observed (arrows).

The physical examination at the patient’s initial visit to our hospital revealed marked motor paralysis of the right upper limb. During voluntary movement in a standing position, right shoulder abduction was limited to 35 degrees and flexion to 50 degrees, while right elbow flexion was restricted to 20 degrees (Figure 3a). In passive movement, both the right shoulder and right elbow exhibited a full range of motion without evidence of joint contracture. In the supine position, voluntary movement was preserved throughout the full range for both joints. Accordingly, the muscle strength of right shoulder abduction, flexion, and right elbow flexion corresponded to Level 2 on the MMT scale.

The patient reported pain extending from the right shoulder to the right upper arm. Muscle strength in the right hand’s interosseous muscles was mildly reduced (MMT Level 4), while the left upper limb and both lower limbs exhibited normal muscle strength. Mild sensory hypalgesia was present from the lateral aspect of the right forearm to the right thumb and index finger. Deep tendon reflexes were diminished in the right upper limb (biceps, triceps, and brachioradialis), whereas reflexes in the left upper limb and both lower limbs were normal. Marked muscle atrophy was observed around the right shoulder, with pronounced atrophy of the deltoid, supraspinatus, and infraspinatus muscles (Figure 3b arrows).

### 2.3. HAL Treatment for the Shoulder Joint

Right shoulder movement treatment using the shoulder HAL commenced 41 months from the onset of C5 palsy (Figure 1). Prior to initiation of HAL therapy, the patient underwent conventional rehabilitation consisting of passive range of motion (ROM) exercises for the upper limb joints, electrical muscle stimulation applied to the affected muscles, assisted active exercises (either self-assisted or therapist-assisted), and upper-limb weight-bearing training aimed at functional use. Despite these conventional modalities, no clinically meaningful improvement in motor function was observed. In contrast, HAL therapy differed in that it enabled active assistive training driven by residual voluntary electromyogram (EMG) signals, thereby providing interactive biofeedback and promoting patient-initiated movements.

**Figure 4:**
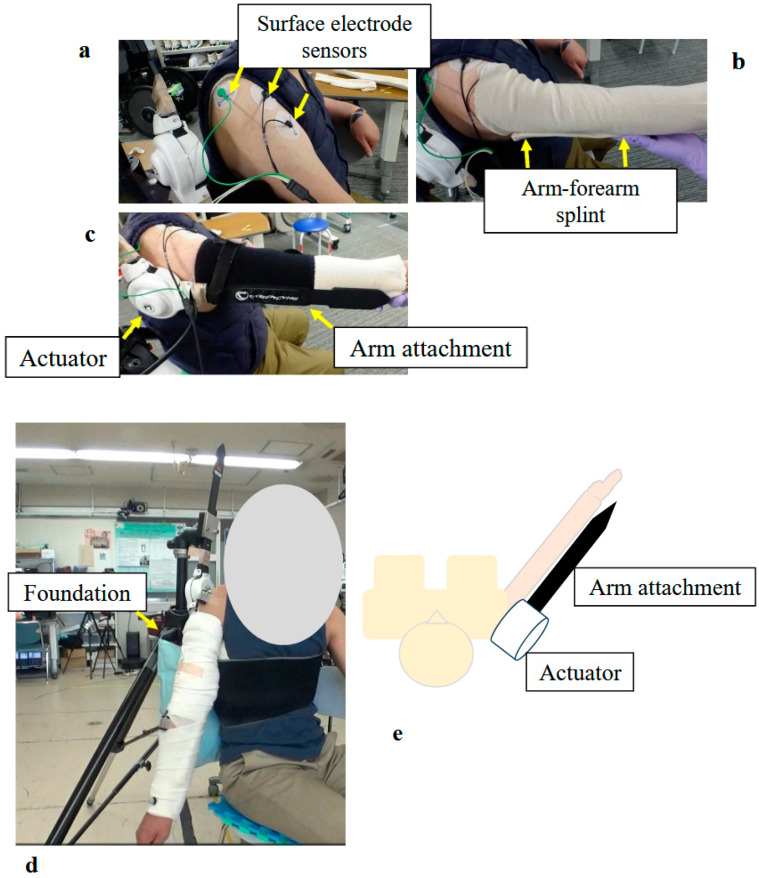
Photographs and Schematic Illustrating the Structure and Donning Procedure of the Shoulder Joint HAL: (**a**) Surface electrodes were attached to the middle and anterior fibers of the participant’s deltoid muscle. (**b**) The upper arm and forearm were wrapped with a securing dressing stockinet, and an arm–forearm splint was applied. (**c**) The arm attachment was fixed to the upper arm and forearm. Special care was taken to align the actuator with the rotational center of the shoulder joint. (**d**) Fully assembled shoulder joint HAL, including the base unit. (**e**) Schematic diagram of the shoulder joint HAL.

**Figure 5:**
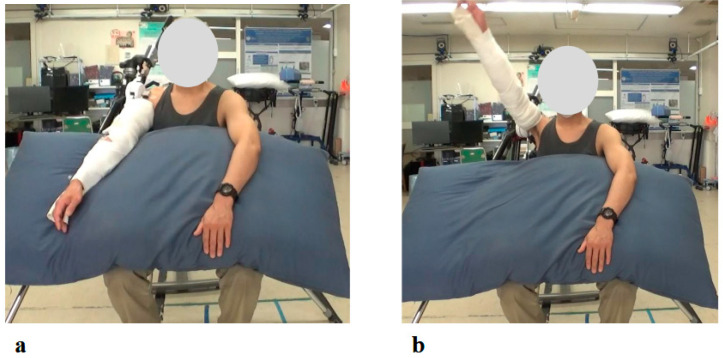
Photographs from the Initial Shoulder Joint HAL Training Session: (**a**) Starting position before arm elevation with the HAL device attached. Training was performed in a seated position, with arm elevation within the scapular plane. (**b**) Maximum arm elevation assisted by the HAL. The shoulder joint HAL was configured to support flexion up to 120 degrees, and full elevation to this maximum angle was successfully achieved during the initial session.

**Figure 6:**
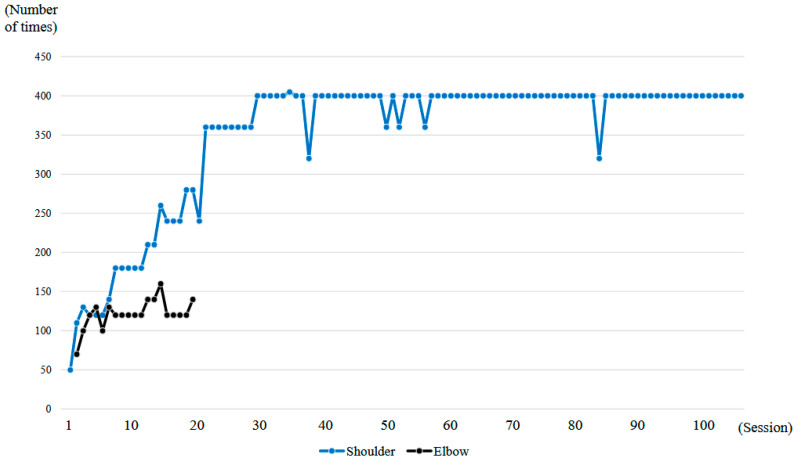
Graph showing the number of right upper limb elevations and right elbow flexions performed per session during the HAL treatment period.

The shoulder HAL was adapted to the shoulder motion as described previously [10]. The actuator of the device was located right below the acromion so that the HAL was aligned with the shoulder joint (Figure 4). In order to generate appropriate shoulder motion, surface electrodes of the device were positioned on the participant’s middle/anterior fibers of the deltoid to detect bioelectric signals triggering arm elevation. Gain parameters, which translate muscular activity into torque of the actuator, were adjusted at the start of every session to provide the most comfortable control to the user. Patients were in a sitting position, and the HAL was attached in a way that elevation could occur in the scapular plane (Figure 5). This plane, parallel to the scapular body and defined as 30–45° from the frontal plane, was chosen as it minimizes scapular movement and the risk of joint and surrounding structures’ injuries. To protect the skin and maintain the arm position, the upper limb was covered with a securing dressing stockinet and a forearm splint. Subsequently, the arm attachment was affixed to the upper arm, and an elastic bandage covered the dressing, securing the entire upper limb in a neutral forearm and elbow extension position (Figure 4). The shoulder HAL treatment was principally conducted once a week, and ultimately 106 sessions were performed (Figure 6). Each training session, lasting for 30–45 min, involved a maximum of 400 repetitions of shoulder elevation exercises utilizing the shoulder assistive robot while the patient was seated. The HAL shoulder flexion angle was set to 120 degrees. Throughout the exercises, either an operator or a therapist managed the controller and provided support for the device. The number of repetitions per session was adjusted based on patient factors, including fatigue, motivation, and pain. For the shoulder HAL, surface electrodes were placed on the deltoid as the primary agonist muscle, and training consisted of repeated shoulder elevation. The HAL detected voluntary EMG activity of the deltoid and provided assistive torque to facilitate elevation. The downward phase primarily reflected relaxation-assisted lowering rather than active extension; however, the training principle was to augment deltoid activation and strengthen it through repeated elevation.

### 2.4. HAL Treatment for the Elbow Joint

**Figure 7:**
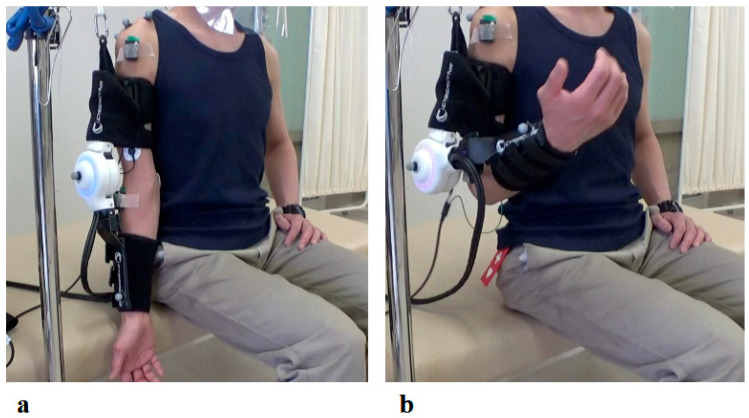
Photographs of Elbow Joint HAL Treatment: (**a**) Starting position with the HAL device attached. Training was conducted in a seated position, with the elbow initially in full extension. (**b**) Maximum elbow flexion assisted by the HAL. The device was configured to provide assistance up to the predetermined target flexion angle.

The right elbow HAL training was conducted in a total of 19 sessions from the second session to the twentieth session, following the shoulder HAL training, as part of the 106 total HAL training sessions (Figure 6). The duration of one treatment session was about 30 min, including fitting and evaluation. Elbow flexion exercises were performed a maximum of 160 times with breaks, for about 15–20 min. Surface electrodes were attached to the right biceps and triceps. During treatment with the elbow joint HAL, the patient was seated at the end of the bed, and treatment was carried out with the arms resting on a table (Figure 7). The upper limb single joint HAL has bioelectric signals balancing capability, and is able to adjust the detected flexion and extension signals balance by means of computer processing, enabling deliberate changes in the elbow flexion and extension assist torque. This capability was employed to set elbow flexion emphasis [9]. For the elbow HAL, electrodes were placed on both the biceps and triceps to reduce co-contraction and promote smooth reciprocal activation. The HAL continuously adjusted assistive torque in real time based on EMG signals from both agonist and antagonist muscles, thereby facilitating coordinated flexion–extension movements [11].

### 2.5. Measurement

We assessed muscle strength in the right deltoid muscle using an MMT and a hand-held dynamometer (HHD, u-Tas F-1, Anima Corp, Tokyo, Japan). HHD testing was performed with a slight modification of the method reported by Andrews et al. [12]. Deltoid HHD testing was performed with the patient standing, with the elbow extended at 0 degrees perpendicular to the shoulder joint (shoulder flexion 0) and the forearm in a neutral position. The HHD sensor was positioned 10 cm distal from the acromion, and the trunk and upper limb were secured with a belt; the patient was instructed to abduct his shoulder, and the shoulder abduction force was measured quantitatively. HHD testing was performed immediately before each treatment. Additionally, the motor subscore of the Functional Independence Measure (FIM) was assessed [13].

### 2.6. Date Collection and Analysis

During each session, muscular activity of the shoulder muscles (upper fibers of the deltoid and trapezius) was recorded using the Trigno™ Lab Wireless Surface EMG system (Delsys Inc., Boston, MA, USA) at 2000 Hz. The deltoid and trapezius were selected as the primary focus of the study. Because this investigation involved an unusually long intervention period, EMG analysis was not conducted during the very first HAL session. Instead, data from the early phase (5th session) were used, at which point the patient had already adapted to the training and safety was ensured, thereby providing a more reliable baseline measurement. The late phase (105th session) was chosen to represent the endpoint of long-term intervention. At both time points, EMG was recorded under no-HAL and HAL-assisted conditions, allowing evaluation of both device-related modulation of muscle activity and longitudinal neuromuscular adaptations across the intervention.

## 3. Results

A total of 106 HAL training sessions were conducted for this case: combined shoulder and elbow HAL training was performed from session 2 to session 20, while shoulder HAL training alone was administered in session 1 and from session 21 to session 106. HAL training was generally carried out once a week, and the training period extended over 21 months (Figure 6).

Throughout the entire duration of the HAL training, no apparent complications occurred. The training was not discontinued at any point due to shoulder pain, fatigue, or other related issues.

**Figure 8:**
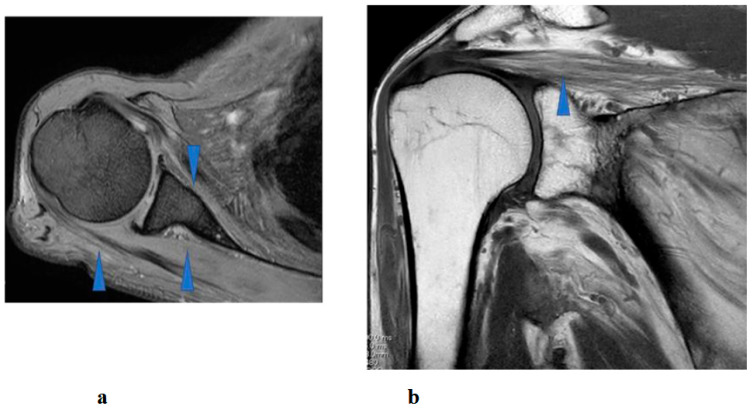
Right shoulder T1 and T2-weighted magnetic resonance imaging showed atrophy and fatty infiltration of several musculus, including supraspinatus, deltoideus, and subscapularis (arrow) on coronal (**a**) and sagittal (**b**).

Right shoulder T1- and T2-weighted MRI before intervention showed atrophy and fatty infiltration of several musculus, including supraspinatus, deltoideus, and subscapularis on sagittal and coronal. Following 8 months and 14 months started HAL training. MRI showed no change in atrophy and fatty infiltration of the supraspinatus, deltoideus, and subscapularis. This assessment was backed by a radiologist (Figure 8).

**Figure 9:**
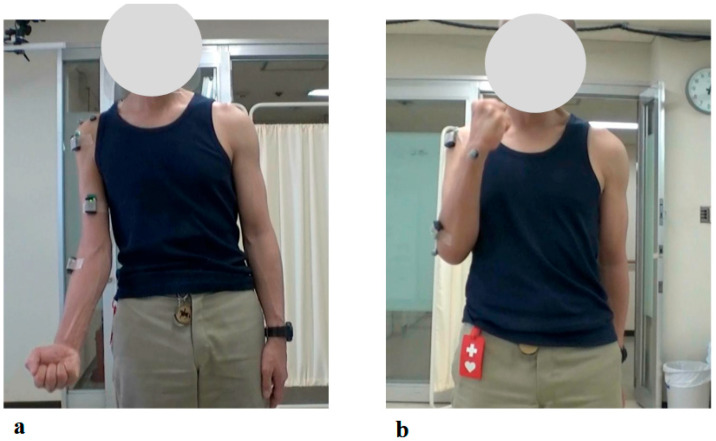
Photographs of Maximum Active Elbow Flexion During the 1st and 19th HAL Treatment Sessions: (**a**) Just before the 1st HAL session, active elbow flexion was limited to 20 degrees. (**b**) By the 19th HAL session, the flexion angle had improved to 145 degrees, with successful active elbow flexion.

At the time of the first training session, the right elbow ROM in a weight-bearing position showed a maximum active flexion angle of 20 degrees (MMT = 2) (Figure 9a). With continued elbow flexion-extension training using the elbow HAL, the flexion angle increased relatively rapidly, reaching 90 degrees by the sixth session (MMT = 3) (1.5 months after the start of HAL training). The flexion angle continued to improve, reaching 145 degrees by the seventeenth session, and this angle was maintained thereafter (Figure 9b).

**Figure 10:**
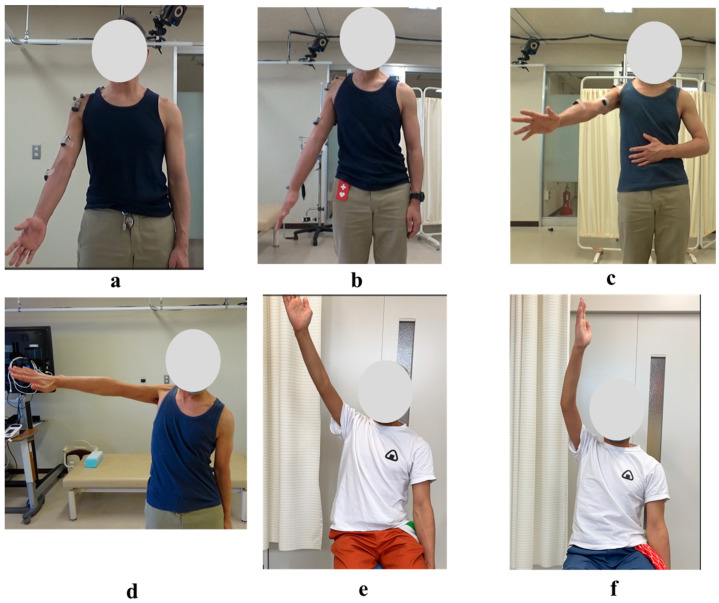
Photographs of Maximum Active Abduction of the Right Shoulder During and After the HAL Training Period: (**a**) Just before the 1st HAL session. (**b**) Just before the 40th HAL session. (**c**) Just before the 60th HAL session. (**d**) Just before the 106th HAL session. (**e**) Nineteen months after completion of HAL training. (**f**) Twenty-five months after completion of HAL training.

**Figure 11:**
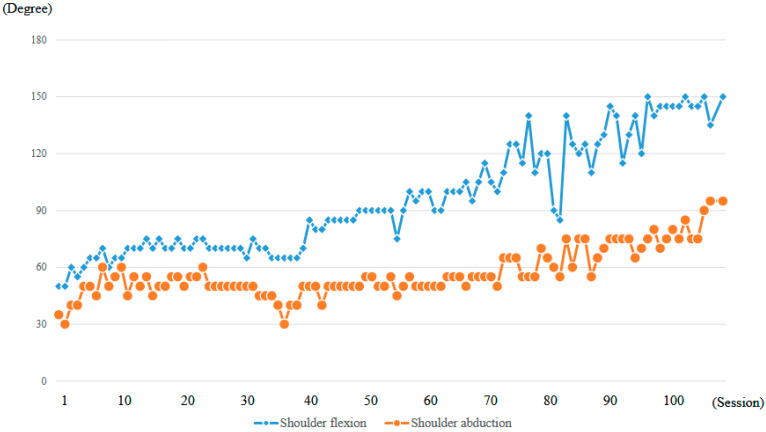
Graph depicting the progression of maximum active shoulder flexion and abduction angles recorded at each session throughout the HAL treatment period.

At the time of the first training session, ROM of the right shoulder in a weight-bearing position was 50 degrees in flexion and 35 degrees in abduction (MMT = 2) (Figure 10a, Table 1). With the progression of right upper limb elevation training using the shoulder HAL, the shoulder flexion angle initially increased steadily, reaching 70 degrees by the eighth session (Figure 11). However, the angle plateaued thereafter despite continued training. Around the 41st session, the flexion angle began to increase again, reaching 90 degrees by the 49th session (8 months after the start of HAL training). The angle continued to improve, ultimately reaching 150 degrees by the final (106th) session (21 months after the start of HAL training) (Figure 11, Table 1).

The right shoulder abduction angle also initially increased steadily with the progression of right upper limb elevation training using the shoulder HAL, reaching 60 degrees by the eighth session (Figure 11). However, the angle plateaued thereafter and did not exceed 60 degrees despite continued training (Figure 10b,c). It was not until the 72nd session that the angle exceeded 60 degrees for the first time, after which it gradually increased. By the 104th session (20 months after the start of HAL training), the abduction angle finally reached 90 degrees (MMT = 3). At the final (106th) HAL session (21 months after the start), the abduction angle was 95 degrees (Figure 10d and Figure 11, Table 1).

Even after the completion of HAL training, the right shoulder ROM continued to improve. At the final follow-up (25 months after the end of HAL training, 46 months after its initiation), the flexion angle had reached 165 degrees and the abduction angle 170 degrees (Figure 10e,f; Table 1).

**Figure 12:**
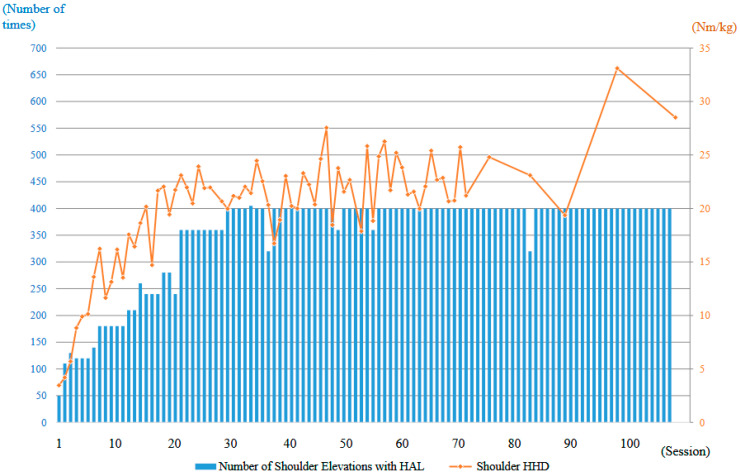
Graph illustrating the progression of right deltoid muscle strength, as measured using a handheld dynamometer (HHD), along with the number of right upper limb elevations performed per session during the HAL treatment period.

The strength of the right deltoid muscle, as measured using an HHD, was 3.5 Nm/kg at the first training session. With continued right upper limb elevation training using the shoulder HAL, the deltoid strength increased markedly in the early phase, reaching 20 Nm/kg by the 16th session (2 months after the start of HAL training). Although the strength continued to improve thereafter, the rate of increase was slower compared to the initial phase. At the final (106th) session of HAL training (21 months after initiation), the deltoid strength reached 28.5 Nm/kg (Figure 12). Regarding the FIM, the motor subscore remained 87 both before and after HAL therapy, with the primary dependent items being eating, bathing, and dressing. Therefore, no measurable improvement in the FIM score was observed.

**Figure 13:**
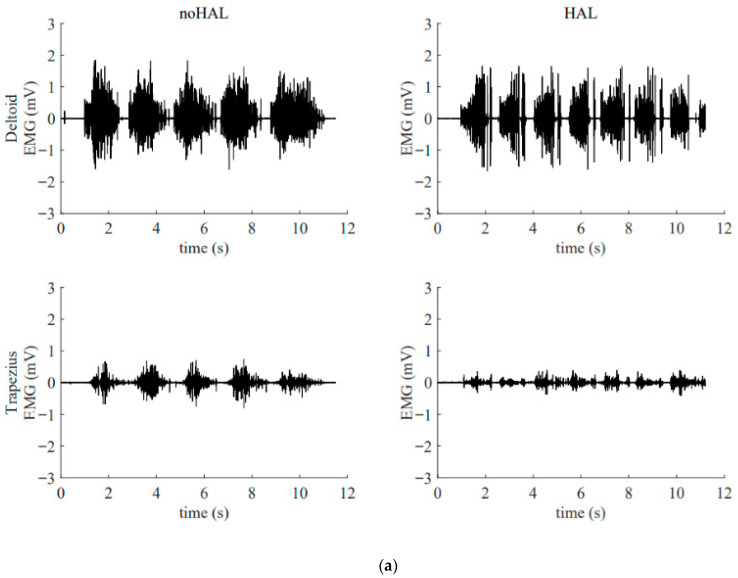

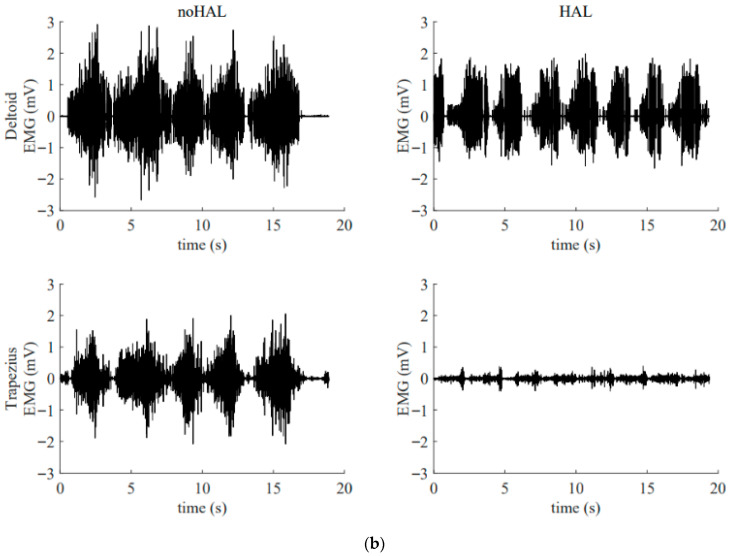
Surface electromyogram of the deltoid and trapezius muscles without and with the HAL during an early session (**a**) and a late session (**b**). (**a**) Data from the 5th HAL session. During this session, the right shoulder joint abduction angle was 50° and the flexion angle was 60°. (**b**) Data from the 105th HAL session. During this session, the right shoulder joint abduction angle was 95° and the flexion angle was 135°.

Figure 13 illustrates the electromyography profiles of the deltoid and trapezius muscles during an early session (Figure 13a) and a late session (Figure 13b). Without the HAL, the muscular activity of the deltoid and trapezius in the late session was markedly increased compared with that in the early session. With the HAL, muscular activity of the trapezius was reduced compared with the condition without the HAL. This reduction was particularly evident in the late session.

## 4. Discussion

Numerous studies have reported on postoperative C5 palsy following cervical spine surgery. However, considerable variability exists among reports regarding the time course and proportion of patients in whom recovery occurs after the onset of palsy. Furthermore, the relationship between recovery from palsy and impairment in activities of daily living (ADL) has not been clearly established [1,2,3,4].

We believe that this inconsistency arises from the insufficient clinical definition of C5 palsy and its recovery. In many studies, C5 palsy is defined as a decrease of at least one grade in the strength of the deltoid or biceps muscle on MMT compared with the preoperative level [1,2,3,4]. Under this definition, a patient whose MMT grade declines from 5 to 4 is categorized in the same group as one whose grade declines from 5 to 2. However, the degree of ADL impairment in a patient with MMT grade 2 is significantly greater than in a patient with grade 4, and the need for therapeutic intervention also differs markedly.

Similarly, the definition of recovery from C5 palsy in many studies is also limited. Recovery is often defined as an improvement of at least one MMT grade in the strength of the deltoid or biceps from the paralyzed state [1,2,3,4]. Furthermore, cases in which muscle strength recovers to MMT grade 5 are categorized as “complete recovery,” whereas those with improvement to MMT grade 4 or below are categorized as “incomplete recovery”. However, when comparing a patient whose muscle strength improves from MMT grade 4 to 5 with one whose strength improves from grade 2 to 3, both represent a one-grade improvement, but the latter may gain significantly more in terms of ADL function. In addition, the former is classified as having “complete recovery,” while the latter is considered “incomplete recovery”. We find it problematic that the patient classified as having “incomplete” recovery may, in fact, achieve greater functional benefit in daily life than the one labeled as “complete”.

To address the aforementioned issues, Saadeh et al. reviewed previous studies and proposed a new set of definitions for C5 palsy and its recovery [14]. Specifically, they defined mild C5 palsy as MMT grade 4, moderate C5 palsy as grade 3, and severe C5 palsy as grade 2 or below. Regarding recovery, they classified cases as complete recovery when muscle strength improved to MMT grade 5, sufficient recovery when it improved to grade 4, and useful recovery when it improved to grade 3. We support the definitions proposed by Saadeh et al. According to their classification, the present case was categorized as severe C5 palsy from onset until the initiation of HAL training, and had improved to moderate C5 palsy by the end of the 106 HAL training sessions. This degree of recovery corresponds to what they define as useful recovery.

Based on the above definitions, Saadeh et al. conducted a detailed investigation and report on the recovery of C5 palsy in their own clinical cases [15]. According to their findings, among 30 patients classified as having severe C5 palsy two weeks postoperatively, 12 recovered to useful strength (MMT grade ≥3) within three months after surgery. An additional six patients recovered between 3 and 6 months, and three more between 6 and 12 months postoperatively. At 12 months, however, nine patients (30%) remained in the severe C5 palsy category. These results suggest that the likelihood of achieving useful recovery in cases of severe C5 palsy decreases as time from surgery progresses. Although this study did not evaluate recovery beyond 12 months postoperatively, when considered in conjunction with other reports, the probability of achieving useful recovery after 12 months appears to be extremely low. In the present case, the patient remained in a state of severe C5 palsy for 41 months postoperatively—a remarkably prolonged duration—and it is highly likely that, without HAL intervention, the chance of achieving useful recovery would have been negligible.

Foraminotomy has been proposed as a surgical treatment for C5 palsy. Several studies have reported that performing prophylactic foraminotomy during cervical spine surgery reduces the incidence of postoperative C5 palsy [1,16]. In addition, there are reports of cases in which foraminotomy was performed secondarily after the onset of C5 palsy, resulting in neurological improvement [1,17]. In the present case, a second surgery involving foraminotomy was performed with the intent to treat the C5 palsy that developed after the initial procedure. However, the palsy worsened following the foraminotomy. This clinical course suggests that the C5 palsy in this case was particularly treatment-resistant.

Interestingly, despite the patient’s functional improvements in ROM and muscle strength, serial MRI assessments performed at 8 and 14 months after the initiation of HAL therapy demonstrated no appreciable reversal of atrophy or fatty infiltration in the supraspinatus, deltoid, or subscapularis muscles. This finding suggests that the observed recovery was not primarily attributable to structural restoration of muscle bulk, but rather to neuromuscular mechanisms such as enhanced recruitment of residual motor units, cortical reorganization, and motor learning facilitated by interactive biofeedback training with HAL [18]. In cases of chronic peripheral neuropathy with longstanding muscle weakness (MMT grade 2 persisting for years), spontaneous recovery is generally considered limited. Therefore, the improvement observed in this patient may have resulted from hypertrophy of partially innervated muscle fibers, increased efficiency of motor unit recruitment, and central neuroplastic adaptations compensating for peripheral denervation [19]. Moreover, by providing real-time biofeedback and suppressing maladaptive compensatory patterns, HAL therapy may have facilitated more physiologic movement strategies, thereby amplifying the functional gains.

A notable feature of the patient’s recovery was the biphasic trajectory of shoulder ROM. While ROM plateaued during the early phase of HAL-assisted training (approximately the first 40 sessions), deltoid muscle strength demonstrated marked improvement. This pattern may reflect a sequential process in motor recovery: an initial phase of neurophysiological adaptation and enhanced recruitment of residual motor units, followed by a later phase of motor learning, cortical reorganization, and functional consolidation, which contributed to subsequent ROM gains [18,19].

The patient was also instructed to perform structured home exercises, including deltoid and rotator cuff strengthening, three sets daily for approximately six months after the initiation of HAL therapy. Therefore, the secondary phase of ROM improvement likely reflects a combination of continued adaptation during HAL sessions and guided home exercise, rather than unsupervised activity.

These findings suggest that HAL therapy may facilitate stepwise neuromuscular recovery, highlighting the potential importance of prolonged, iterative training and complementary home exercises for optimizing functional outcomes in chronic upper-limb palsy. Such insights may inform future rehabilitation protocols integrating robotic-assisted therapy with patient-guided home exercises.

In the present case, conventional rehabilitation was administered over an extended period; however, it was ineffective, and the patient remained in a state of severe C5 palsy. In many previous reports, general physical therapy has been prescribed for patients who develop C5 palsy following cervical spine surgery. However, there is currently no clear evidence regarding the extent to which such rehabilitation contributes to neurological recovery [1,2,3,4].

Taken together, the evidence suggests that in this case, the likelihood of achieving useful recovery from severe C5 palsy—persisting for 41 months postoperatively—was extremely low with conventional rehabilitation alone. However, by initiating HAL therapy at 41 months after onset and continuing for a total of 106 sessions over 21 months, the patient ultimately achieved useful recovery. This outcome indicates that HAL therapy may exert a therapeutic effect distinct from that of conventional rehabilitation. This raises the question of what underlies the distinct therapeutic mechanism of HAL.

It is important to emphasize that although HAL is a type of robotic device, its therapeutic effect is not merely due to power assistance. The mechanism of action of HAL is explained by the theory of interactive biofeedback (iBF) [7]. This theory posits that the use of HAL facilitates the establishment of a closed-loop system between the brain and the musculoskeletal system. In patients with paralysis, even if the brain issues motor commands, the intended movement cannot be executed accurately. However, when wearing HAL, the device supports the movement in accordance with the brain’s intention, enabling the patient to perform the desired action. This movement is then perceived by the brain, which in turn facilitates the generation of subsequent motor commands. Through this mechanism, the use of HAL induces changes in brain activity. Our previous studies using functional MRI and functional near-infrared spectroscopy (fNIRS) have demonstrated such changes [20,21].

In addition, it is important to emphasize the role of errorless motor learning in HAL training. When a patient with deltoid paralysis attempts to elevate the upper limb without assistance, excessive activation of the trapezius muscle often occurs, resulting in an abnormal shoulder movement known as a “shrugging motion,” which represents a compensatory strategy. Repeated training under such conditions can lead to the consolidation of these compensatory patterns in the brain, potentially impeding the recovery of normal motor function. As demonstrated in the present case (Figure 13), HAL can suppress such compensatory movements, thereby promoting more physiologically appropriate motion patterns [10,11]. This suppression is considered another key factor contributing to functional recovery in patients with paralysis.

In the present case, HAL training was effective even in a chronic and treatment-resistant case of C5 palsy, initiated 41 months after onset. We previously reported a case of severe C5 palsy in which HAL treatment was started 7 months after onset [12]. Compared with that case, both the number of HAL sessions and the duration of training required to achieve useful recovery were markedly greater in the present case. Specifically, in the 7-month case, deltoid muscle strength improved to MMT grade 3 after 12 sessions of HAL training over a 6-month period. In contrast, the present case required 104 sessions over 20 months to achieve the same level of recovery. Furthermore, we have also reported on five cases of severe C5 palsy in which HAL training was initiated in the early postoperative period (within 21 days of onset). In those early-intervention cases, the effects of HAL training appeared earlier and more prominently, suggesting that earlier initiation of HAL therapy may lead to more rapid and significant functional recovery.

Based on these findings, it is likely preferable to initiate HAL training before C5 palsy becomes chronic. However, the optimal timing for initiating HAL therapy after the onset of C5 palsy remains to be determined and warrants further investigation. It is necessary to accumulate data by applying HAL training at various stages following onset. Ideally, a prospective comparative study including a control group receiving only conventional rehabilitation without HAL would provide more definitive conclusions regarding the effectiveness and appropriate timing of HAL intervention.

A limitation of this case report is the absence of upper-limb-specific validated functional measures. While general assessments were performed, they may not fully capture subtle, clinically meaningful changes in hand and arm function. In future studies, we plan to incorporate standardized upper-limb assessments, such as the Disabilities of the Arm, Shoulder, and Hand (DASH) questionnaire or the Shoulder Pain and Disability Index (SPADI), to more accurately evaluate functional improvements.

## 5. Conclusions

In this case, a long-term rehabilitation program combining shoulder and elbow HAL training successfully improved severe postoperative C5 palsy—present for 41 months—to the level of useful recovery. This experience suggests the potential effectiveness of HAL therapy for treatment-resistant postoperative C5 palsy.

## Figures and Tables

**Table 1:** Changes in Maximum Active Abduction and Flexion Angles of the Right Shoulder Joint Across Evaluation Time Points During and After HAL Treatment.

	Angles of the Right Shoulder Joint	
	Abduction (°)	Flexion (°)
1st HAL session (41 m)	35	50
106th HAL session (62 m)	95	150
8 m after 106th HAL session (70 m)	85	140
15 m after 106th HAL session (77 m)	90	135
19 m after 106th HAL session (81 m)	160	150
25 m after 106th HAL session (87 m)	170	165

The parentheses ( ) indicate the time following the 1st surgery. m: month.

## Data Availability

The original contributions presented in this study are included in the article. Further inquiries can be directed to the corresponding author.
